# Intercropping grapevine with *Solanum nigrum* enhances their cadmium tolerance through changing rhizosphere soil microbial diversity

**DOI:** 10.3389/fmicb.2025.1537123

**Published:** 2025-03-07

**Authors:** Changbing Pu, Ziyao Huang, Xuefeng Jiang, Jiawei Zhu, Lijin Lin, Xiaoli Zhang, Hui Xia, Dong Liang, Jin Wang, Xiulan Lv

**Affiliations:** College of Horticulture, Sichuan Agricultural University, Chengdu, China

**Keywords:** fruit tree, hyperaccumulator, intercropping, rhizosphere soil properties, microbial community

## Abstract

**Introduction:**

Cadmium (Cd), a toxic heavy metal, has increasingly impacted vineyard soils and grapevine (*Vitis vinifera*) production in recent years. Intercropping with the hyperaccumulator plant *Solanum nigrum* has emerged as a promising strategy to improve soil health and increase plant resilience to the Cd-contaminated soil.

**Methods:**

This study investigated the effects of intercropping grapevine with *S. nigrum* (IntVVSN) on the soil enzyme activity and microbial community.

**Results:**

Compared with the monocultures of *S. nigrum* and grapevine, IntVVSN increased the activities of soil sucrase, soil urease, and soil cellulase, and decreased the activities of soil amylase, and soil neutral phosphatase. The microbial community in IntVVSN showed higher abundances of beneficial groups such as Acidobacteriota, Actinobacteriota, and Chloroflexi. These groups were involved in the metal detoxification and nutrient cycling, indicating their potential role in enhancing Cd tolerance. Kyoto Encyclopedia of Genes and Genomes (KEGG) pathway enrichment analysis revealed the distinct metabolic adaptations in IntVVSN under Cd-contaminated soil, with significant upregulation of pathways related to the secondary metabolite synthesis, carbohydrate metabolism, glycan biosynthesis, nucleotide metabolism, and protein processing. The changes in microbial composition, along with the enhanced nutrient cycling indicated by increased soil enzyme activities, suggest a healthier and more resilient soil environment. This, in turn, contributes to improved Cd tolerance in grapevines.

**Conclusion:**

This study highlights the phytoremediation potential of *S. nigrum* intercropping, which promotes sustainable agricultural practices in Cd-contaminated soil by improving plant growth and resilience to heavy metal stress.

## 1 Introduction

With the rapid industrialization and extensive use of agricultural inputs, heavy metal contamination has emerged as a pressing environmental issue in China (Mamut et al., 2023). Orchard soil is particularly affected, increasing public awareness and concern (Wang Q. et al., 2015; Afra et al., 2022). Cadmium (Cd), a widespread and highly toxic heavy metal, has increasingly polluted vineyard soil and grapevine in recent years (Mirzaei et al., 2020; Yang et al., 2022). The high toxicity of Cd suppresses the soil microbial activity, disrupts the nutrient cycling and reduces the soil fertility. It also inhibits the growth of plant roots and shoots, reduces crop yields and introduces Cd into the food chain, where it bioaccumulates and endangers human and animal health (Meng et al., 2022; Zhu et al., 2024). Thus, sustainable and effective remediation strategies are urgently needed to address Cd-contaminated soil.

Intercropping, the practice of cultivating multiple crops together, improves the yields, soil health, and pest control while enhancing the land use efficiency through beneficial ecological interactions (Brooker et al., 2015; Li et al., 2023a). Specifically, intercropping hyperaccumulator plant with conventional crop helps to remediate the heavy metal-contaminated soil (Zhang D. et al., 2022). For example, intercropping the Cd/zinc (Zn) hyperaccumulator plant *Sedum plumbizincicola* with *Moso bamboo* significantly enhanced heavy metal removal from contaminated soils (Bian et al., 2021). Similarly, intercropping rice with the Cd-accumulator plant *Solanum nigrum* reduced the Cd uptake in rice (Kama et al., 2023), and intercropping with the arsenic (As) hyperaccumulator *Pteris vittata* reduced the Cd content in maize (*Zea mays*) grains by 66.7% in multi-metal-contaminated soil (Zeng et al., 2024). In addition to metal uptake, rhizospheric interactions among intercropped species improve the soil health and restructure microbial communities, supporting the sustainable soil remediation (Huang et al., 2023). For example, the intercropping of Chinese cabbage with *S. nigrum* increased the rhizosphere soil microbial diversity and altered the microbial community composition (Li et al., 2023b). Similarly, intercropping *P. vittata* and *Sedum alfredii* increased the soil bacterial diversity, with relatively high abundances of *Lysobacter*, *Massilia*, and *Arthrobacter* in their respective rhizospheres (Wang X. et al., 2022). These changes in microbial communities are often associated with the improved nutrient cycling, enhanced stress tolerance, and increased stability of soil ecosystems, which collectively contribute to more effective remediation (Huang et al., 2023; Li et al., 2023b; Wang X. et al., 2022). Therefore, intercropping heavy metal hyperaccumulators with crops can reduce the heavy metal uptake in crops and affect the soil microbial diversity. However, the intercropping of different plant species has various effects on these aspects.

Grape (*Vitis vinifera*), an economically valuable vine native to Western Asia, is cultivated globally for fruit production, particularly for wine, raisins, and juice (Wang C. et al., 2022). However, industrialization and intensive fertilizer and pesticide use have increased the Cd contamination in vineyards (Girotto et al., 2016; Santana et al., 2015). In previous study, intercropping with *S. nigrum* reduced the Cd accumulation in grape shoots by approximately 29.8%, highlighting its phytoremediation potential in Cd-contaminated soil (Hu et al., 2019). However, the specific effects of intercropping *S. nigrum* with grapevine in Cd-contaminated soil on the rhizosphere soil microbial communities remain unclear. In this study, we evaluated the effects of intercropping *S. nigrum* with grapevine in Cd-contaminated soil, with a focus on changes in rhizosphere soil microbial diversity. The aim of this study was to determine whether intercropping could change the rhizosphere soil microbial diversity and provide a reference for the safe production of grapes in Cd-contaminated areas.

## 2 Materials and methods

### 2.1 Materials

The grapevine variety used in this experiment was ‘Summer Black’. Its branches were collected from the vineyard of Sichuan Agricultural University (33°33′N, 103°38′E) in December 2022, and stored in sand. The branches with a length of 15 cm were cut into trays filled with the perlite in March 2023, and the trays were placed in a greenhouse under the conditions of Li et al. (2022) until the new shoots grew to 15 cm.

Seeds of *S. nigrum* were collected from the farm of Sichuan Agricultural University in October 2022, air dried, and stored at 4°C. In April 2023, *S. nigrum* seeds were sown in the soil. When the seedlings of *S. nigrum* grew to a height of 10 cm, they were transplanted.

The soil sample used in this experiment was fluvo-aquic soil, which was collected from the farm of Sichuan Agricultural University. The basic physicochemical properties of the soil samples were pH 7.55, organic matter content 12.09 g/kg, total nitrogen content 0.49 g/kg, total phosphorus content 0.58 g/kg, total potassium content 11.55 g/kg, alkaline hydrolyzable nitrogen content 42.95 mg/kg, available phosphorus content 12.39 mg/kg, available potassium content 41.33 mg/kg, and total Cd content 0.23 mg/kg.

### 2.2 Experimental design

The experiment was conducted at Chengdu Campus of Sichuan Agricultural University, Sichuan Province, China, from April to June 2023. In April 2023, the soil samples were air-dried and treated as described by Lin et al. (2020). The weight of 3.0 kg soil samples were filled in each plastic pot with dimensions of 20 cm (height) × 21 cm (diameter). Then, Cd(NO_3_)_2_·4H_2_O was added to the soil to achieve a final soil Cd concentration of 5 mg kg^−1^ to prepare the Cd-contaminated soil (Hu et al., 2019). The potted soil was kept moist for 1 month and subsequently mixed for further use. In May 2023, *S. nigrum* (10 cm in height) and grapevine (15 cm in height of new shoots) seedlings were transplanted into pots. The experiment was divided into three treatments: (1) monoculture of *S. nigrum* (MonSN), (2) monoculture of grapevine (MonVV), and (3) intercropping of grapevine with *S. nigrum* (IntVVSN). Each treatment was conducted in triplicate (three pots). Two plants were planted in each pot. For the monoculture, two *S. nigrum* seedlings or grapevines were planted in each pot. For the intercropping, one *S. nigrum* seedling and one grapevine were planted in each pot. Pots were randomly arranged to establish a completely randomized design, and watered in a timely manner to keep the soil moist until the plants were harvested.

### 2.3 Soil sample collection

In July 2023, after 45 days of plant growth, the rhizosphere soil samples were collected via the shaking root method. One part of each soil sample was stored at 4°C for the analysis of soil enzyme activities. Other soil samples were immediately frozen with liquid nitrogen and transported to Beijing BioMarker Technologies Co., Ltd. (Beijing, China) for microbial community sequencing.

### 2.4 Determination of soil enzyme activity

Soil enzyme activities, including soil amylase (G0318W), soil sucrase (G0302W), soil catalase (G0303W), soil neutral phosphatase (G0306W), soil urease (G0301W), and soil cellulase (G0308W), were measured via the corresponding assay kits (Grace Biotechnology, Suzhou, China) according to the manufacturer’s instructions.

### 2.5 Analysis of soil microbial diversity

#### 2.5.1 DNA extraction and polymerase chain reaction amplification

Total genomic DNA was extracted from MonSN, MonVV, and IntVVSN samples via the T Guide S96 Magnetic Soil/Stool DNA Kit (Tian gen Biotech, Beijing, China) according to the manufacturer’s instructions. The quality and purity of DNA were examined via agarose gel electrophoresis and a NanoDrop 2000 UV–Vis spectrophotometer (Thermo Scientific, Wilmington, United States), respectively. The amplified products referred to the 16S rRNA gene, and amplified using the primers 338F (ACTCTACGGGGAGGCAGCA) and 806R (GGACTACHVGGGTWTCTAAT). The PCR amplification was carried out with an initial denaturation step at 95°C for 5 min, followed by 20 cycles of denaturation at 95°C for 30 s, annealing at 50°C for 30 s, and extension at 72°C for 40 s, with a final extension at 72°C for 7 min. The amplified products were purified with an Omega DNA purification kit (Omega Inc., Norcross, GA, United States) and quantified via a Qsep-400 (BiOptic, New Taipei City, Taiwan, ROC). The amplicon library was paired-end sequenced (2 × 250) on an Illumina NovaSeq 6000 platform.

#### 2.5.2 Bioinformatic analysis

The Non-chimeric Reads with more than 97% similarity thresholds were allocated to one operational taxonomic unit (OTU) via USEARCH. Taxonomic annotation of the OTUs/ASVs was performed via the naive Bayes classifier in QIIME2 (Bolyen et al., 2019) via the SILVA database (Quast et al., 2012) with a confidence threshold of 70%. Alpha diversity (α diversity) was determined via QIIME2 software to identify the complexity of the species diversity of each sample. Beta diversity (β diversity) calculations were performed via principal coordinate analysis (PCoA) to assess the diversity in samples for species complexity using the R Programming Language. The heat maps were generated using the R Programming Language. For each bacterial taxon, the abundance values across the different samples were Z-score standardized using the scale function in the R Programming Language, which normalized the data to have a mean of 0 and a standard deviation of 1. The 16S rRNA feature sequences were aligned to the reference sequences from the IMG database using PICRUSt2 (v2.3.0) (Srivastava et al., 2024). A phylogenetic tree was built to identify the closest species to each feature. Gene profiles were predicted based on related species’ gene types and abundance. These predictions were integrated with KEGG pathway data to infer the microbial community’s metabolic pathways (Parks et al., 2014). PICRUSt2 compared feature sequences to a phylogenetic tree for species annotation and utilized IMG microbiome data to predict functional gene composition. The generated abundance table was standardized to account for differences in 16S rRNA gene copy numbers. KEGG information for each feature was then used to calculate the functional gene abundance and its distribution across pathways.

### 2.6 Statistical analysis

All experimental data were analyzed via SPSS 22.0, and visualizations were created with GraphPad Prism. Significant differences were evaluated through one-way ANOVA followed by Duncan’s multiple range test (*p* < 0.05).

## 3 Results

### 3.1 Rhizosphere soil enzyme activity

**Figure 1:**
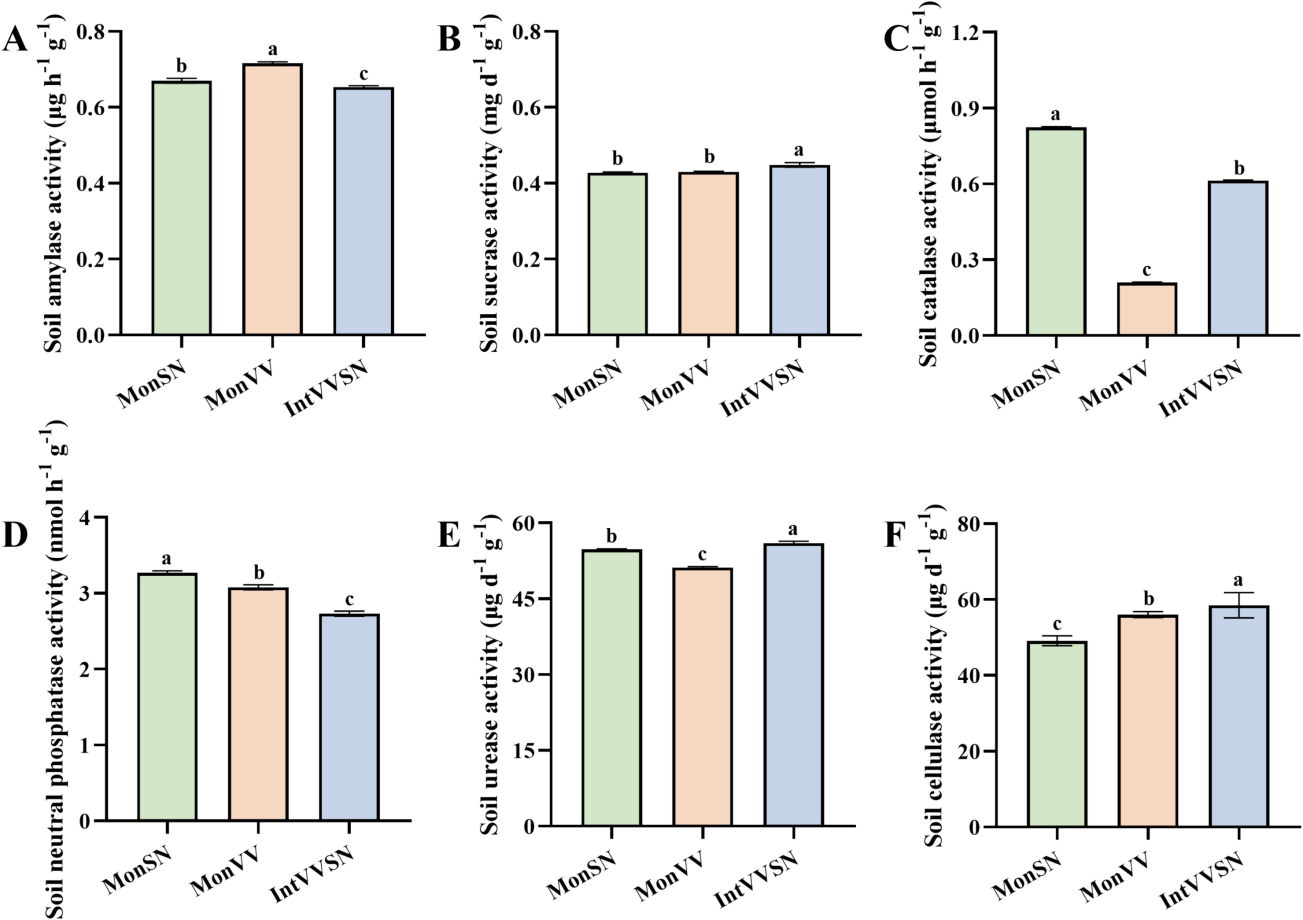
Enzyme activities in rhizosphere soil under MonSN, MonVV, and IntVVSN treatments. Different lowercase letters indicate significant differences among the treatments (Duncan’s multiple range test, *p* < 0.05). **(A)** Soil amylase activity; **(B)** soil sucrase activity; **(C)** soil catalase activity; **(D)** soil neutral phosphatase activity; **(E)** soil urease activity; **(F)** soil cellulase activity. MonSN, monoculture of *S. nigrum*; MonVV, monoculture of grapevine; IntVVSN, intercropping grapevine with *S. nigrum*.

Compared with monocultures of grapevine and *S. nigrum*, intercropping of grapevine with *S. nigrum* changed the activities of six soil enzymes in Cd-contaminated soil (Figure 1). These enzymes, which include amylase, sucrase, catalase, neutral phosphatase, urease, and cellulase, play critical roles in soil nutrient cycling, organic matter decomposition, and overall soil health (Daunoras et al., 2024). The soil amylase activity in IntVVSN was the lowest among the three treatments, with the order of MonVV > MonSN > IntVVSN (Figure 1A), suggesting that IntVVSN might reduce starch degradation in the rhizosphere. The soil sucrase activity in IntVVSN was greater than that in MonSN or MonVV (Figure 1B). There was no significant difference in soil sucrase activity between MonSN and MonVV, indicating that intercropping might increase the hydrolysis of sucrose, although the effect was relatively small compared with that of monocultures. The soil catalase activity was ranked as MonSN > IntVVSN > MonVV (Figure 1C). These findings suggest that the monoculture of *S. nigrum* was more effective at maintaining oxidative stress responses, whereas intercropping resulted in intermediate catalase activity, which might reflect a different balance in soil oxidative processes. The soil neutral phosphatase activity in IntVVSN was the lowest among the three treatments, with the order of MonSN > MonVV > IntVVSN (Figure 1D), indicating that intercropping might inhibit the mineralization of organic phosphorus. Compared with MonSN and MonVV, IntVVSN increased the soil urease activity, and the order of soil urease activity was IntVVSN > MonSN > MonVV (Figure 1E), suggesting that IntVVSN enhanced the nitrogen cycling, which could increase the nitrogen availability in the rhizosphere, particularly in Cd-contaminated soil. Finally, IntVVSN also increased the soil cellulase activity compared with MonSN and MonVV (Figure 1F). The order of soil cellulase activity was IntVVSN > MonVV > MonSN, suggesting that intercropping could promote the cellulose degradation in the soil by increasing the organic matter decomposition and nutrient cycling.

### 3.2 Analysis of microbial diversity and community structure

The samples from the monoculture soils of MonSN, MonVV, and IntVVSN were processed and sequenced, generating a total of 720,022 paired-end reads. After quality control, 644,360 clean reads were retained across all the samples, with an average of 71,596 clean reads per sample (Supplementary Table S1). These reads were clustered into operational taxonomic units (OTUs), resulting in an average of 3,629–3,747 OTUs in MonSN, 3,293–3,574 OTUs in MonVV, and 3,542–3,629 OTUs in IntVVSN (Supplementary Table S2).

**Figure 2:**
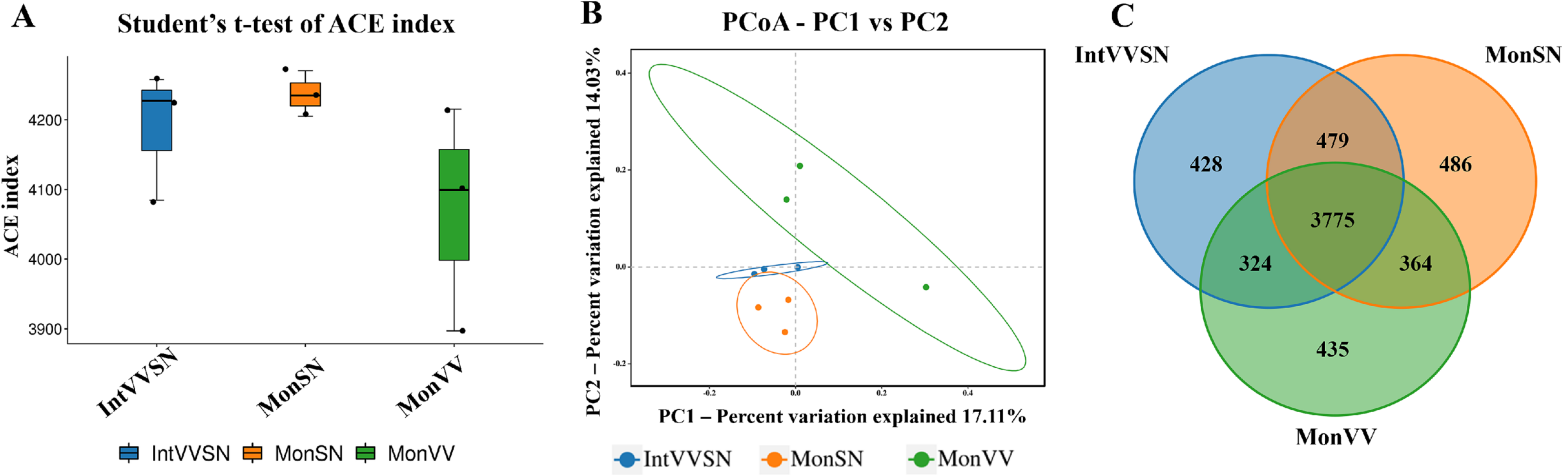
Microbial diversity and community structure in MonSN, MonVV, and IntVVSN soils. **(A)** Boxplot of ACE indices for microbial communities; **(B)** PCoA of microbial communities; **(C)** Venn diagram of shared and unique OTUs. MonSN, monoculture of *S. nigrum*; MonVV, monoculture of grapevine; IntVVSN, intercropping grapevine with *S. nigrum*.

Alpha diversity analysis, as assessed by the ACE index, revealed no significant differences in microbial richness among MonSN, MonVV, and IntVVSN (Figure 2A; Supplementary Table S3). PCoA further revealed differences in the microbial community composition among the three treatments, with the first principal coordinate (PC1) explaining 17.11% of the variation and the second (PC2) explaining 14.03% (Figure 2B; Supplementary Table S4). IntVVSN and MonSN clustered more closely, whereas MonVV exhibited a distinct community structure. The Venn diagram revealed that 3,775 OTUs were shared across the three treatments, with 428, 486, and 435 unique OTUs in IntVVSN, MonSN, and MonVV, respectively (Figure 2C). These results underscored the shared and unique microbial communities shaped by different cropping systems.

### 3.3 Analysis of bacterial community composition and abundance

Supplementary Table S5 presents the taxonomic diversity of the bacterial communities across the different cropping systems. A total of 33 phyla, 86 classes, 256 orders, 499 families, 929 genera, and 1,067 species were identified under the three cropping systems. IntVVSN and MonSN showed comparable diversity at each taxonomic level, whereas MonVV had slightly lower diversity, particularly at the class, order, and family levels. These results suggested that IntVVSN and MonSN supported a more diverse microbial community in the rhizosphere than MonVV did.

**Figure 3:**
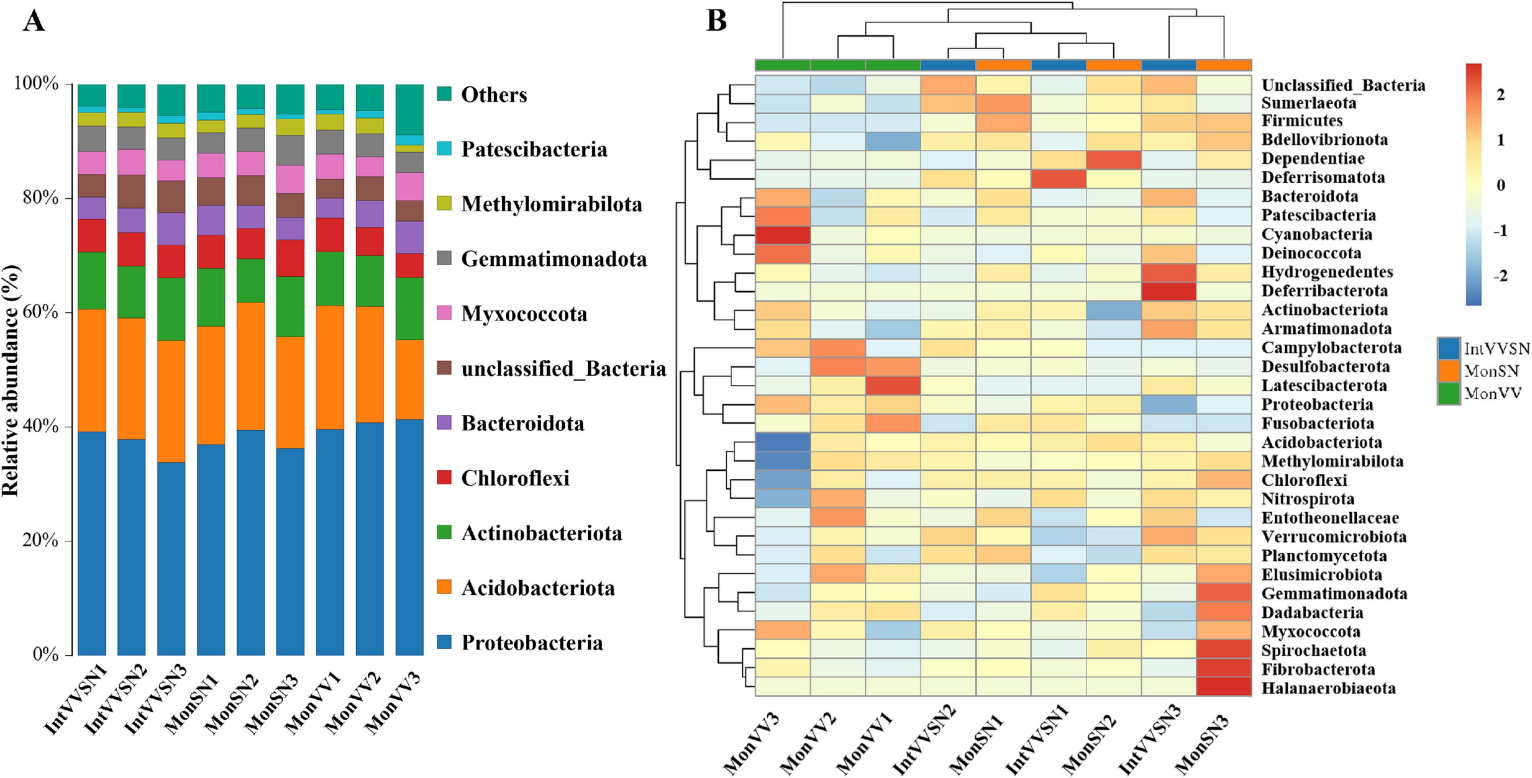
Relative abundance and clustering of bacterial phyla in IntVVSN, MonSN, and MonVV. **(A)** Relative abundance of dominant bacterial phyla; **(B)** Heatmap of bacterial phyla abundance patterns. MonSN, monoculture of *S. nigrum*; MonVV, monoculture of grapevine; IntVVSN, intercropping grapevine with *S. nigrum*. Serial numbers (1, 2, and 3) represent three repetitions.

The relative abundance and clustering of bacterial phyla in the rhizosphere soils of the three cropping systems were shown in Figure 3. Across all the cropping systems, Proteobacteria, Acidobacteria, and Actinobacteria were the dominant phyla, with Proteobacteria consistently exhibiting the highest relative abundance (Figure 3A; Supplementary Table S6). Acidobacteriota and Actinobacteria were particularly enriched in IntVVSN and MonVV, suggesting that their adaptability to the specific conditions of these cropping systems, such as Chloroflexi, Bacteroidota, Myxococcota, and Gemmatimonadota, likely contributed to the overall community composition but played secondary roles.

Heatmap analysis revealed distinct microbial profiles among the cropping systems (Figure 3B). The samples of MonVV formed a distinct cluster, suggesting that a unique microbial community structure was influenced by the monoculture of grapevine. In contrast, the samples of MonSN and IntVVSN were intermixed, indicating that *S. nigrum* influenced the rhizosphere microbial community similarly, whether it was grown alone or intercropped. Campylobacterota, Desulfobacterota, Latescibacterota, and Proteobacteria were more abundant in MonVV, reflecting favorable conditions for these groups in the monoculture of grapevine. Moreover, Firmicutes, Sumerlaeota, Deferrisomatota, Dependentiae, and Bdellovibrionota were notably enriched in MonSN and IntVVSN, suggesting that their preference for rhizosphere conditions were associated with *S. nigrum*. This similarity between the MonSN and IntVVSN treatments highlighted the consistent impact of *S. nigrum* on shaping microbial communities in the rhizosphere.

**Figure 4:**
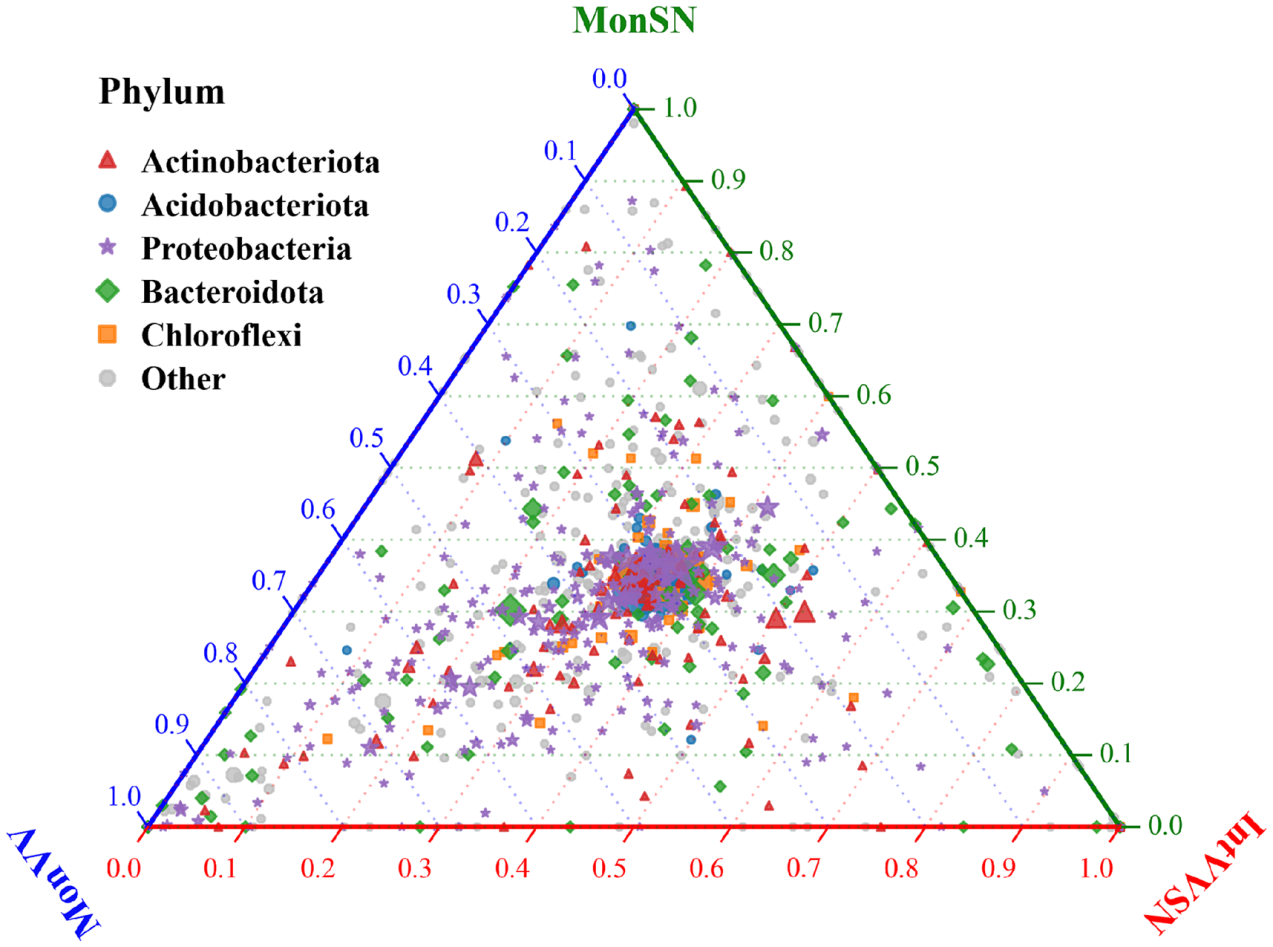
The relative abundance distributions of major microbial phyla. MonSN, monoculture of *S. nigrum*; MonVV, monoculture of grapevine; IntVVSN, intercropping grapevine with *S. nigrum*.

The ternary plot illustrated the relative abundance distribution of major microbial phyla across MonSN, MonVV, and IntVVSN (Figure 4). The results revealed that Proteobacteria and Bacteroidota were predominantly distributed in the central region of the plot, indicating that their relative abundances were fairly balanced across the three treatment groups, with no clear bias toward any specific group. Actinobacteria was positioned closer to the blue and green axes, suggesting relatively greater abundances of MonSN and MonVV. Acidobacteriota and Chloroflexi were evenly distributed across the plot, indicating that their abundances were consistent across all the treatment groups.

### 3.4 KEGG pathway enrichment analysis

**Figure 5:**
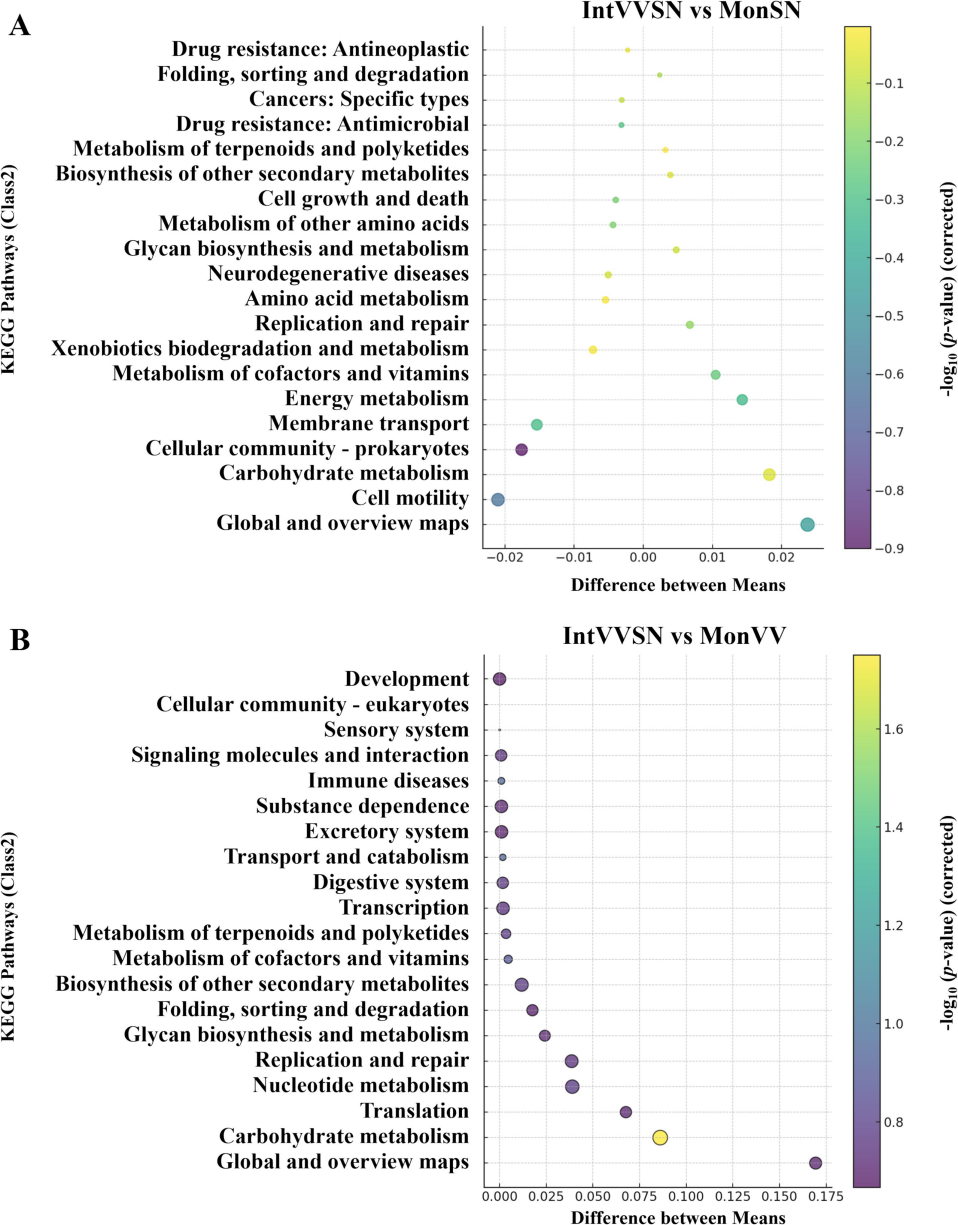
KEGG pathway enrichment bubble plots comparing the intercropping treatment with the single-planting treatments under Cd stress. The x-axis represents the difference in the mean relative abundance (%) of each pathway between the two groups, and the y-axis represents the KEGG pathways (Class 2). The size of each bubble corresponds to the absolute difference in mean relative abundance, and color intensity indicates statistical significance as measured by −log_10_ (*p* value corrected). **(A)** Comparison between IntVVSN and MonSN; **(B)** IntVVSN and MonVV. MonSN, monoculture of *S. nigrum*; MonVV, monoculture of grapevine; IntVVSN, intercropping grapevine with *S. nigrum*.

To investigate the effects of intercropping on the Cd stress mitigation, we performed a KEGG pathway enrichment analysis in which IntVVSN was compared with MonSN and MonVV (Figure 5). The enrichment profiles revealed significant functional differences in metabolic and stress-response pathways across the different treatments. As shown in Figure 5A and Supplementary Table S7, the most KEGG pathways clustered around the zero point on the x-axis, indicating minimal differences in the relative abundance of general metabolic processes between IntVVSN and MonSN. However, several pathways, particularly those associated with the antioxidant defense, energy metabolism, and secondary metabolite biosynthesis, were significantly enriched in IntVVSN, suggesting that intercropping enhances the specific metabolic functions to mitigate Cd toxicity. Under IntVVSN treatment, energy metabolism pathways, including the glycolysis and the tricarboxylic acid (TCA) cycle, were more active, providing essential energy for cellular activities and repair processes under Cd stress. Additionally, carbohydrate metabolism pathways in IntVVSN appear to contribute to the energy production and antioxidant responses, likely assisting in the reactive oxygen species (ROS) detoxification through glycolytic pathways. In contrast, MonSN was enriched in pathways related to the membrane transport, cellular community-prokaryotes, and cell motility, which facilitated the translocation of substances across cell membranes, increased Cd uptake and contributed to Cd stress management.

The KEGG pathway enrichment analysis also revealed distinct metabolic adaptations to Cd stress in IntVVSN compared with MonVV, with significant differences in pathways related to the secondary metabolite synthesis, glycan biosynthesis, nucleotide metabolism, carbohydrate metabolism, and protein processing (Figure 5B; Supplementary Table S8). Among these pathways, the carbohydrate metabolism pathway presented the most pronounced differences, indicating the substantial metabolic reprogramming in the intercropping system. IntVVSN demonstrated broader and more complex metabolic responses, suggesting that its mixed-species composition provides the enhanced adaptability and resilience under Cd stress, potentially offering a more versatile defense mechanism than the single-species MonVV system.

## 4 Discussion

Cd contamination has become a growing issue in orchard ecosystems and affects soil quality, tree growth, fruit safety, and overall biodiversity (Mirzaei et al., 2020; Yang et al., 2022). Intercropping with hyperaccumulator plants, such as *S. nigrum*, has emerged as an effective strategy for remediating the Cd-contaminated soil (Wang S. et al., 2015). By planting these hyperaccumulator plants, the Cd bioavailability in the soil can significantly reduce, thereby minimizing the Cd uptake by neighboring crops (Zhang D. et al., 2022). Previous studies have shown that intercropping with *S. nigrum* effectively reduced the Cd accumulation in grape shoots and promoted its growth (Hu et al., 2019). Therefore, our study investigated the comparative effects of a grapevine-*S. nigrum* intercropping system (IntVVSN) and monocultures (MonVV and MonSN) on enhancing the Cd phytoremediation in vineyard soil. Our findings indicate that IntVVSN can create a more resilient rhizosphere environment under Cd stress. This resilience was evidenced by modifications in soil enzyme activity, shifts in microbial community structure, and the enrichment of key metabolic pathways, highlighting the potential of intercropping for sustainable Cd management in vineyard ecosystems.

Soil enzyme activity indicates the capacity of enzymes in soil to catalyze biochemical reactions, serving as a key indicator of soil health and biological activity, and it plays a vital role in organic matter decomposition, nutrient cycling, and soil structure formation (Wallenstein et al., 2009; Han et al., 2022). Soil sucrase breaks down sucrose into glucose and fructose, providing a carbon source for soil microbes and indicating the intensity of soil organic carbon decomposition and cycling (Tang et al., 2022). Soil urease produces ammonia and carbon dioxide, providing an effective nitrogen source for plants and playing a crucial role in soil nitrogen cycling to increase the soil nitrogen supply capacity (Rana et al., 2021). Soil cellulase, which is produced mainly by microbes such as fungi and bacteria, is essential for the decomposition of cellulose in soil and organic matter (Mokale Kognou et al., 2022). Soil catalase reduces toxicity, protects soil microbes, and reflects soil redox conditions and microbial activity, and serves as an indicator of soil quality and potential Cd contamination effects (Liu et al., 2024). Soil amylase activity hydrolyzes starch into simpler sugars, providing carbon sources for microbes and indicating soil organic matter turnover and microbial health (Li et al., 2011). Soil neutral phosphatase activity converts organic phosphorus into bioavailable inorganic phosphate, which plays a key role in phosphorus cycling and soil fertility (Cao et al., 2022). The intercropping of apple and marigold led to a significant increase in the activities of urease, phosphatase, sucrase, and cellulase in rhizosphere soils (Xue et al., 2023). Compared with monoculture tea and walnut forests, intercropped forests presented greater sucrase activity but significantly lower peroxidase activity (Bai et al., 2022). However, in our study, compared with monocultures of grapevine and *S. nigrum*, intercropping of grapevine with *S. nigrum* increased the activities of soil sucrase, urease, and cellulase, which was consistent with peach-Morchella intercropping (Song et al., 2021), intercropping of chestnut trees (*Castanea mollissima*) in tea (*Camellia sinensis*) (Ma et al., 2017), and intercropping of *Vicia sativa* in young kiwifruit orchards (Wang et al., 2021). The observed increases in enzyme activities can attribute to intercropping-induced changes in microbial diversity and activity driven by the provision of abundant substrates and the alteration of microbial community composition through plant–plant interactions. These mechanisms stimulate the production of extracellular enzymes involved in carbon and nitrogen cycling, thereby improving nutrient availability, alleviating soil stress, and enhancing organic matter decomposition and overall soil health. Conversely, the activities of soil amylase and neutral phosphatase in the intercropping of grapevine with *S. nigrum* were lower than the monocultures of grapevine and *S. nigrum*, whereas the activity of catalase in the intercropping of grapevine with *S. nigrum* was among the monocultures of grapevine and *S. nigrum*. The reduction in the activities of amylase and phosphatase may be explained by competition between grapevine and *S. nigrum* for carbon and phosphorus resources, as well as shifts in root exudation patterns that influence microbial functionality. Catalase activity is an intermediate, reflecting a balanced oxidative stress environment. Overall, this study demonstrated that intercropping, as a mechanism of species diversification, selectively modulates soil enzyme activity, supporting ecosystem productivity and soil health (Cong et al., 2015; Power, 2010).

Microbial diversity and community structure are central to the health and functionality of the rhizosphere, particularly under stress conditions (Wooliver et al., 2022; Gupta et al., 2022). Intercropping enhances the soil microbial diversity by increasing plant diversity and promoting root interactions, improving heavy metal bioavailability and plant absorption (Li et al., 2020; Zhang et al., 2024; Li et al., 2024). Integrating intercropping with microbiome research can optimize soil remediation strategies and increase heavy metal bioremediation efficiency (Wang X. et al., 2022). Our results revealed that the microbial community composition of IntSNVV was more similar to that of MonSN, which is similar to that of the intercropping system of the arsenic hyperaccumulators *P. vittata* and maize (Zeng et al., 2024). Additionally, intercropping did not change the Cd concentrations in corn and soybean but affected the soil available Cd levels, and the dominant phyla in the rhizospheres of both soybean and corn were Proteobacteria, Chloroflexi, Acidobacteria, Actinobacteria, and Firmicutes (Li et al., 2022). In the intercropping group, the relative abundances of Proteobacteria, Acidobacteria, and Actinobacteria increased slightly, whereas those of Firmicutes and Planctomycetes decreased, indicating that garlic may affect microbial community diversity (Hussain et al., 2021). In this study, IntVVSN resulted in a more diverse microbial community than MonVV, with increased abundances of Acidobacteriota, Actinobacteriota, and Chloroflexi. Notably, Acidobacteriota and Actinobacteriota are known to thrive in nutrient-limited and metal-stressed environments, suggesting that the microbial community in IntVVSN is well adapted to Cd-stressed conditions (Janssen, 2006).

In Acidobacteriota phylum, microorganisms in *Acidobacteriaceae* family demonstrated strong adaptability under heavy metal stress (Supplementary Table S9). These microorganisms likely mitigate metal toxicity by adjusting their metabolic pathways and binding metal ions (Zhang et al., 2023). Within Acidobacteriales order, certain microbial populations presented the increased abundance in IntVVSN, suggesting that these microbes enhance soil health through metal chelation and metabolic adaptation. Notably, the abundance of *Acidobacteriaceae* and *Koribacteraceae* families increased significantly in IntVVSN, indicating their competitive advantage in Cd-contaminated environments. These microorganisms improve soil quality by decomposing organic matter and mineralizing organic substances, thus reducing the adverse effects of heavy metals on plant growth (Zhang M. et al., 2022). The increase in *Candidatus_Koribacter* further supports their role in metal stress tolerance and soil health promotion (Schmidt et al., 2025). Additionally, the abundance of *unclassified Acidobacteriales* species significantly increased, contributing to the alleviation of Cd toxicity to plants.

In Actinobacteria phylum, microorganisms in Actinobacteria class exhibit strong adaptability in Cd-contaminated soils (Supplementary Table S9). These microbes, which are capable of decomposing organic matter and participating in nitrogen cycling, contribute to soil health (Xie et al., 2022). Within Actinobacteriales order, bacteria from the *Nocardiaceae* family grew more abundantly in Cd-contaminated soils. Microorganisms from the *Nocardiaceae* and *Streptomycetaceae* families are highly resistant to metals, effectively degrading harmful substances and adsorbing metal ions, thereby reducing the bioavailability of Cd in the soil (Timková et al., 2018). The abundance of *Nocardia* and *Streptomyces* species increases, reinforcing their role in mitigating Cd toxicity to plants via metal chelation, and certain *Streptomyces* species increase soil nutrient availability and microbial diversity, further supporting the metal remediation process (Selatnia et al., 2004).

In Chloroflexota phylum, microorganisms in Chloroflexia class thrive in high-metal-concentration environments, demonstrating strong ecological adaptability in metal-contaminated soils (Supplementary Table S9) (Freches and Fradinho, 2024). Within Chloroflexales order, certain microorganisms showed an increased abundance in IntVVSN, indicating their ability to adapt to Cd contamination and effectively remove or transform heavy metals (Zou et al., 2024). Microorganisms in *Chloroflexaceae* family promoted metal transformation and precipitation through bioreduction, alleviating Cd toxicity. The enrichment of *Dehalococcoides* and other genera from *Chloroflexaceae* family further suggests their role in purifying Cd from the soil via enhanced metabolic pathways (Bandara et al., 2022). *Dehalococcoides* and other species can reduce harmful substances through dechlorination or reduction reactions, lowering the bioavailability of heavy metals and protecting plants from Cd-induced damage (Wang J. J. et al., 2022).

To analyze the functional pathways of the soil microorganisms, we compared the microbial gene sequences with those in the KEGG database (Parks et al., 2014). KEGG pathway enrichment analysis revealed distinct metabolic adaptations in IntVVSN under Cd stress, with significant upregulation of pathways related to secondary metabolite synthesis, carbohydrate metabolism, glycan biosynthesis, nucleotide metabolism, and protein processing. The pronounced changes in carbohydrate metabolism, in particular, indicate that IntVVSN engaged in significant metabolic reprogramming to meet the energy demands and antioxidative requirements imposed by Cd stress. The enrichment of carbohydrate metabolism, such as glycolysis and the TCA cycle pathways, indicated the increased energy production, which is critical for cellular repair and detoxification under Cd stress. Additionally, carbohydrate metabolism pathways in IntVVSN contribute to antioxidant defense through glycolytic pathways, playing a crucial role in detoxifying the ROS generated by Cd stress (Luo et al., 2024). Similarly, the secondary metabolites, such as phenolics, flavonoids, and alkaloids, are known for their role in chelating heavy metals and scavenging ROS, suggesting that intercropping enhances the production of these compounds to mitigate Cd toxicity (Vitelli et al., 2024). The broader metabolic responses observed in IntVVSN suggest that, compared with monoculture systems, intercropping systems have greater flexibility and resilience. By promoting the synthesis of stress-related compounds, IntVVSN likely improved the stress tolerance of grapevine plants, mitigating Cd-induced damage. This study highlighted the synergistic relationship between *S. nigrum* and grapevine, demonstrating how specific plant–plant and plant-microbe interactions in the intercropping system effectively enhanced heavy metal uptake while simultaneously promoting plant growth.

## 5 Conclusion

This study demonstrated that IntVVSN enhanced the Cd phytoremediation in vineyard soils. Compared with the monocultures, IntVVSN resulted in elevated soil sucrase, soil urease, and soil cellulase activities. Additionally, the microbial diversity in the intercropped rhizosphere was closely mirrored that of MonSN, indicating that *S. nigrum* strongly influenced the microbial community composition and potentially enhanced the Cd tolerance. This intercropping system presents a promising approach for both the Cd remediation and vineyard soil health management. To build on these findings, future research should focus on the transcriptomic and metabolomic analyses to identify the related transcription factors to confirm their roles in Cd tolerance and phytoremediation.

## Data Availability

The datasets presented in this study can be found in online repositories. The names of the repository/repositories and accession number(s) can be found below: https://www.ncbi.nlm.nih.gov/sra/PRJNA1193995.
